# Mediation of the stigma in the influence of negative symptomatology over recovery in psychosis

**DOI:** 10.1016/j.ijchp.2021.100220

**Published:** 2021-02-01

**Authors:** Nuria Ordóñez-Camblor, Mercedes Paino, Eduardo Fonseca-Pedrero, Juan Pablo Pizarro-Ruiz

**Affiliations:** a Departament of Health Sciences, Faculty of Health Sciences, University of Burgos, Spain; b Departament of Psychology, Faculty of Psychology, University of Oviedo, Spain; c Departament of Education, University of La Rioja, Spain; d Departament of Education, Faculty of Education, University of Burgos, Spain

**Keywords:** Psychotic disorders, Stigma, Recovery, Negative symptomatology, Descriptive survey study, Trastornos psicóticos, Estigma, Recuperación, Sintomatología negativa, Estudio descriptivo de poblaciones

## Abstract

*Background/Objective*: The interest in recovery processes in psychotic disorders has boosted the necessity of knowledge about the factors that could influence in such recovery. Negative symptomatology and the stigma have been negatively linked to the recovery process in psychosis. The aim of this investigation is to improve the understanding of how the recovery process is affected by negative symptomatology based on the analysis of the mediating effects of the internalized stigma. *Method*: The sample was composed of 114 people that had experienced, at some point in their life, at least one clinically relevant psychotic episode. CAPE-42, STORI and ISMI were used for the evaluation. The macro PROCESS for SPSS was used. The indirect effect was calculated using 10.000 samples of bootstrap for the bootstrap confidence intervals (IC) corrected for bias. *Results*: The results show that the influence of negative symptomatology predicts the stigmatization of the person regarding his disorder. This predicts a negative influence in the recovery process of the psychosis. *Conclusions*: These results back the importance of adding the reduction of the stigma as a specific strategy to improve the recovery process in psychotic disorders.

## Abstract (es)

*Antecedentes/Objetivo*: El interés por los procesos de recuperación en los trastornos psicóticos ha impulsado la necesidad de conocer los factores que podrían influir en dicha recuperación. La sintomatología negativa y el estigma se han relacionado negativamente con el proceso de recuperación de la psicosis. El objetivo de esta investigación es mejorar la comprensión sobre cómo el proceso de recuperación se ve afectado por la sintomatología negativa a partir del análisis de los efectos mediadores del estigma internalizado. *Método*: La muestra estuvo compuesta por 114 personas que habían experimentado, en algún momento de su vida, al menos un episodio psicótico clínicamente relevante. Para la evaluación se usaron el CAPE-42, STORI and e ISMI. Se utilizó el macro PROCESS para SPSS. El efecto indirecto se calculó utilizando 10.000 muestras de bootstrap para los intervalos de confianza (IC) de bootstrap corregidos por sesgo. *Resultados*: Los resultados muestran que la influencia de la sintomatología negativa predice la estigmatización de la persona con respecto a su trastorno, y esto predice una influencia negativa en el proceso de recuperación de la psicosis. *Conclusiones*: Estos resultados respaldan la importancia de sumar la reducción del estigma como estrategia específica para mejorar el proceso de recuperación en los trastornos psicóticos.

The interest in the study of recovery processes in psychosis has increased in the last few years (Lee et al., 2020, Lemos-Giráldez, Vallina-Fernández et al., 2015). The recovery from a psychotic disorder can be understood as a process or as a result (Jacob, 2015). When considered as a result (also called clinic recovery) it implies a binary concept that is constant in everyone and means a reduction or an elimination of the symptom, added to a better working. This idea does not entail the cases in which there is a substantial remission of the symptoms, even though the general process of the illness persists. Neither does it take into account the cases in which a change in the premorbid status (Bellack, 2006); has happened; nor does it consider the subjective part. The reason for this is that it does not evaluate the level of satisfaction the person has with his life.

In contrast, when considered as a process (also called personal recovery), the recovery is not synonymous with healing and, therefore, implies much more than returning to a premorbid state (Jacob, 2015). It focuses on vital satisfaction, hope and contributions to life despite limitations caused by the disorder (Leamy et al., 2011). This varies depending on people and the empirical evidence sets up a series of stages of change, instead of prevalence rates. In this case, qualitative analysis rather than quantitative, is done, there by emphasising recovery as something more than the absence of symptom signs or failure of performance. From this perspective, recovery is possible despite the existence of psychiatric problems (Leonhardt et al., 2017); meaning recovery, not only as a diachronic process, but also as a process in which the goal is not necessarily restoring competences. Instead, the goal is for the individual to live by growing and developing himself (Pemberton & Wainwright, 2014). It would not be a lineal process, on the contrary it would be a spiraling journey, formed by numerous kinds of experiences, like constant relapses and recoveries (Anthony, 1993). The spiraling metaphor, therefore, could be more useful than the idea of lineal stages, since people often return to previous stages before progressing to more advanced ones (Slade, 2009). This increase in interest in the psychosis recovery processes has strengthened the need for knowledge of the factors that could affect such a recovery.

Regarding the factors that complicate recovery the presence of negative symptoms has been linked to a worse recovery process and a low quality of life (García-Álvarez et al., 2014, Gee et al., 2016, López-Navarro et al., 2018, Norman et al., 2018, Wood et al., 2017). Negative symptoms entail a decay of a wide range of basically affective and conative functions, which are very frequent in people with psychosis; approximately 60% show this kind of symptom (Lemos-Giráldez, García-Alvarez et al., 2015). In 10-30% of the cases they have a high magnitude and persistence leading to the deficiency syndrome. They make a clear impact on the occupational, familiar and social functioning of the patient, as well as on general living and health habits (Paz García-Portilla & Bobes, 2013).

There are therefore many investigations which consider negative symptomatology a relevant risk factor in the prediction of the clinical picture influencing the recovery process in a negative light (Lutgens et al., 2017, Lyne et al., 2018). Furthermore, they are little, if at all, sensitive to pharmacological action, and even such symptomatology may worsen (Freudenreich, 2020).

Another factor that contributes to an increase in relapses and hence to a worse recovery is the stigma. Such is the case, that it has become considered one of the obstacles to a more -if not the most- significant recovery (Ho et al., 2018, Pyle et al., 2018). We estimate that around a third of the people with a serious mental disorder show high stigma levels (Yanos et al., 2011). Stigma means the set of attitudes of negative connotations that a social group has in minority sectors which show some kind of distinctive feature; a distinctive feature or "sign" which, identifying it, produces a negative stereotype in the social conscience towards them (Chang et al., 2018, Roca and Crespí, 2013). We can set up a differentiation between the public or social stigma and the internalized or self-stigma in mental disorders. The first one would be a reaction from society to mental illness, whereas the second one would be the reaction of the mental disorder bearer against themself (Corrigan & Watson, 2002). Thus, self-stigma in a mental health context refers to a process by which a person with a serious mental disorder loses the identities previous to the disease and the hope of having new identities (e.g., I as a student or a worker), and adopts the stigmatized point of view held by many community members (e.g., I as dangerous, I as an incompetent) (Corrigan and Watson, 2002, Yanos et al., 2011). Many studies about internalized stigma have found a strong link to self-stigma with low self-esteem, low feeling of self-sustaining, low social support, low hope, postponing the search for treatment, bad adherence to treatment, and subjective low quality of life (Gerlinger et al., 2013, Lien et al., 2018, Livingston and Boyd, 2010, Young and Ng, 2016). At a psychopathological level, stigmatization has been associated with an increase in depressive symptomatology and anxiety, amongst others (Aukst-Margetić et al., 2014, Park et al., 2013, Schrank et al., 2014). In addition, it can contribute to social isolation, in a decrease of access to health services and to increasing a delay in access to treatments (Kular et al., 2019, Mueser et al., 2020, Xu et al., 2016).

In this context, scientific literature shows that negative symptomatology influences directly in a worse recovery from psychosis. However, the hypothesis of the present study intends to find an alternative explanation: the greater presence of negative symptomatology would lead the patient to suffer from an increase in the stigma. This increase would be the one which would lead to a worse recovery process and not the negative symptoms, which so many difficulties mean to a therapeutic approach.

Therefore, the main aim of this study is to improve the understanding of how negative symptomatology affects the recovery process, based on the analysis of the mediating effects of the internalized stigma. Thus, in line agreeing with results shown in other investigations, we hope to confirm that negative symptomatology complicates the recovery from psychosis (hypothesis 1). Nevertheless, our hypothesis suggest that this total effect would become mediated by changes that negative symptomatology causes to the internalized stigma; meaning, that negative symptoms predict the stigma of the people with psychosis (hypothesis 2) and this is the one that influences negatively in the recovery process from psychosis (hypothesis 3), disappearing in the presence of this mediator (the stigma), the direct effect of negative symptomatology on recovery (hypothesis 4) (Figure 1).

**Figure 1:**
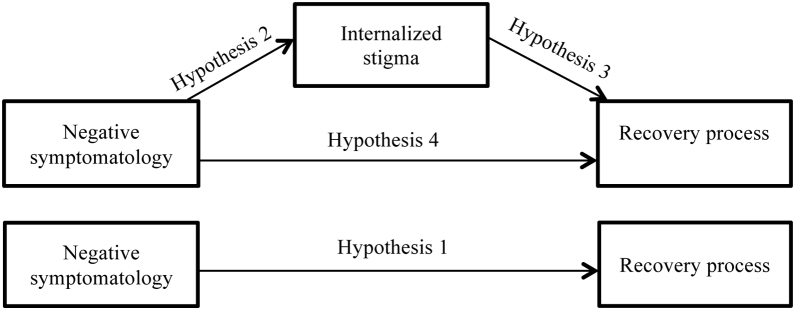
Hypotesized mediation model.

## Method

### Participants

The sample was composed of 114 people that had experienced at least one clinically relevant psychotic episode at some point in their life, independent of the possible triggers for it. The selection of the participants took place in different mental health centres in the Asturias region, Cantabria and Cataluña. All users were in treatment for a spectrum disorder of schizophrenia according to the Diagnostic and Statistical Manual of Mental Disorders (DSM-5, American Psychiatric Association APA, 2013): 71 (62.30%) people had a diagnosis of Schizophrenia, 19 (16.70%) of Brief Psychotic Disorder, 7 (6.10%) of Schizoaffective Disorder, 7 (6.10%) of Bipolar Disorder with Psychotic Symptoms, 5 (4.40%) of Delusional Disorder, 3 (2.60%) of Schizophreniform Disorder and 2 (1.80%) of Schizotypal Disorder. The average number of hospitalizations was 1.48 (*SD* = 1.72) and of psychotic episodes was 2.68 (*SD* = 1.81). Out of the total number of participants, 82 were male (71.90%). The average age was 35.5 years old (*SD* = 9.26), the age range varying between 14 and 52 years old. 62.20% had pharmacological treatment and 36.80% of the sample reported a history of mental illness in the family, and 47.74% reported a personal history of mental disorder.

### Instruments

Community Assessment Psychic Experiences-42 (CAPE-42; Fonseca-Pedrero et al., 2012, Stefanis et al., 2002). This is a self-report that allows to assess psychotic experiences in its affective and non-affective sides in a Likert type of 4 points response format. It is made up of 42 items that assess Positive (20 items), Negative (14 items) and Depressive (8 items) dimensions of psychotic symptoms. The Spanish version of CAPE-42 shows suitable reliability levels and different validity sources that support its use as a measurement of the variation of the psychotic phenotype (Barragan et al., 2011, Obiols et al., 2008, Ros-Morente et al., 2011). Previous studies have used CAPE-42 in clinical samples, in general population and in non-clinical teenagers, showing a suitable psychometric behaviour regarding internal consistency, temporary stability and to different sources of validity (Konings et al., 2006, Ros-Morente et al., 2011, Stefanis et al., 2002).

Stages of Recover Instrument (STORI; Andresen et al., 2006, Lemos-Giráldez, García-Alvarez et al., 2015). It evaluates the recovery stage in which the persons are found, after suffering a psychotic episode. It consists of 50 items, with a Likert type of 5 points response format. The items are assembled in 10 blocks of 5 items each and represent one of the four components of the recovery process: Hope, Identity, Meaning and Responsibility. Each one of these items within each group represents a recovery stage (stage 1: moratorium, stage 2: conscience, stage 3: preparation, stage 4: rebuilding, and stage 5: growth). The stage with the highest total score is considered to be the recovery stage of the person. STORI has shown suitable psychometric properties regarding evidences of validity, as well as test-retest reliability and internal consistency (Andresen et al., 2006, Weeks et al., 2011). This is also one of the four instruments which are suggested to evaluate the recovery routinely in Australian mental health centers (Burgess et al., 2010).

Internalized Stigma of Mental Illness (ISMI; Muñoz et al., 2009, Ritsher et al., 2003). Evaluates the subjective experience of stigma or internalized stigma by those who suffer from mental illness and the impact of social stigma on the experience of everyday life. It includes 29 items, in a Likert of a 4 point, and it entail five subscales: (a) Alienation (6 items): it measures the subjective experience of being less than others or of revealing damaged identity; (b) Approval of the stereotype (7 items): it measures the degree of agreement with the current stereotypes of people with mental illness; (c) Discriminatory experience (5 items): it tries to capture the person's perception of the way he is treated by others; (d) Social isolation (6 items): it evaluates the tendency to socially isolate himself; and (e) Resistance to stigma (5 items): it reflects the experience of resisting or not being affected by the internalized stigma. The higher the score, the bigger the internalized stigma or the one evaluated by each of its factors in the person, except for the subscale "resistance to stigma", in which the highest score indicates less resistance to stigma. The scale shows suitable psychometric properties, alluding to internal consistency, test-retest reliability and the different sources of validity (Ritsher et al., 2003).

The Spanish versions of the questionnaires have followed international guidelines for the development and adaptation of evaluation instruments (Hernández et al., 2020, Muñiz and Fonseca-Pedrero, 2019).

### Procedure

The handling of the sample tests of people with psychosis was carried out individually, during a clinical session and in a room adapted for this purpose. Every participant gave his informed consent to participate in this study voluntarily. The study was presented to the participants as an investigation of early prevention and intervention and the longitudinal follow-up of people with prodomes or a first psychotic episode. We assured them the confidentiality of their answers, as well as the voluntary basis of their participation. No reward for their collaboration in the study was given. The Ethical Committee of Clinical Investigation of the Central University Hospital in Asturias approved this investigation.

### Statistical analysis

Giving an answer to the first objective the macro PROCESS for SPSS developed by Hayes (2018) was used, specifically Model 4, which postulates a mediation model with a mediating variable. This method was used as an analytic strategy to evaluate the indirect effect of negative symptomatology (IV) in recovery from psychosis (DV) through the mediating process of the internalized stigma (Mediator). We calculated the indirect effect using 10.000 samples of bootstrap for the bootstrap confidence intervals (*CI*) corrected for bias. An indirect effect is considered statistically significant if the established *CI* (*CI* at 95%) does not include a 0 value. If the 0 value is included in the *CI*, the null hypothesis establishes that the indirect effect is equal to 0, that is, there is not an association between the involved variables (Hayes, 2018).

Subsequently the indirect effects of the internalized factors of the stigma are studied in order to look into which are the mediators that harm the recovery process the most (Figure 3), and which traditionally have been linked traditionally to the negative symptomatology of psychosis, using a model with five parallel (non-sequential) mediators. We also used the macro PROCESS for SPSS developed by Hayes (2018). As well as in the previous aim, the indirect effect was calculated using 10.000 samples of bootstrap for the bootstrap confidence intervals (*IC*s). The IV (negative symptomatology) and DV (recovery from psychosis) are the same as before, but now there are five parallel mediators which are the dimensions of internalized stigma: Alienation (M1), Approval of the Stereotype (M2), Discriminatory Experience (M3), Social Isolation (M4) and Resistance to Stigma (M5).

## Results

Means and standard deviations of the study variables according to the stage of recovery (Lemos-Giráldez, García-Alvarez et al., 2015) are shown in Table 1.

**Table 1:** Descriptive statistics of the independent variable (Negative CAPE) and the mediators (ISMI) according to the stage of recovery (STORI).

Variable	Stage 1 (*n* = 21)		Stage 2 (*n* = 37)		Stage 3 (*n* = 56)	
	*Mean*	*SD*	*Mean*	*SD*	*Mean*	*SD*
Negative symptomatology	2.37	0.62	1.86	0.57	1.80	0.48
Internalized stigma	2.69	0.37	2.07	0.36	1.91	0.40
Alienation	3.15	0.43	2.33	0.58	2.10	0.54
Approval of the stereotype	2.34	0.47	1.72	0.37	1.66	0.52
Discriminatory experience	2.64	0.54	2.14	0.60	1.86	0.58
Social isolation	2.72	0.59	2.09	0.62	1.90	0.58
Resistance to stigma	2.62	0.44	2.13	0.52	2.09	0.55

First of all, we analysed the influence of negative symptomatology over the recovery stage from psychosis. Regarding Hypothesis 1, the results of the total effect of negative symptomatology (*IV*) on the recovery stage (*DV*) confirm that a bigger presence of negative symptoms complicates the recovery process (*B* = -0.30, *p* = .001).

Regarding Hypothesis 2 it is also confirmed, as expected, that negative symptomatology predicts stigma of people with psychosis (*B* = 0.47, *p* < .0001). Besides, as considered in Hypothesis 3, this level of internalized stigma predicts the recovery from psychosis negatively (*B* = -0.50, *p* < .0001).

The results linked to Hypothesis 4, point at the disappearance of the direct effect of negative symptomatology in the recovery from psychosis (*B* = -0.06, *p* = .458) if one takes into consideration the mediation of the stigma in this link, as was expected in our proposal (Table 2).

**Table 2:** Mediation model: Indirect effect of negative symptomatology on the recovery stage from psychosis through changes in the internalized stigma.

			*B*	*SE*	*p*
Mediating variable model (DV: Internalized stigma)					
Predictor					
Negative symptomatology			.47	2.002	.0001
DV: Recovery process					
Predictors					
Internalized stigma			-.50	0.005	.0001
Negative symptomatology (Direct effect)			-.06	0.120	.458
Total effect					
Negative symptomatology			-.30	0.119	.001
Indirect effect			**B**	**Boot SE**	**Boot 95% CI**
Negative symptomatology →	Internalized stigma →	Recovery process	-.23	0.061	[-.362, -.123]

*Note.* Standardised regression coefficients and completely standardized indirect effect.

**Figure 2:**
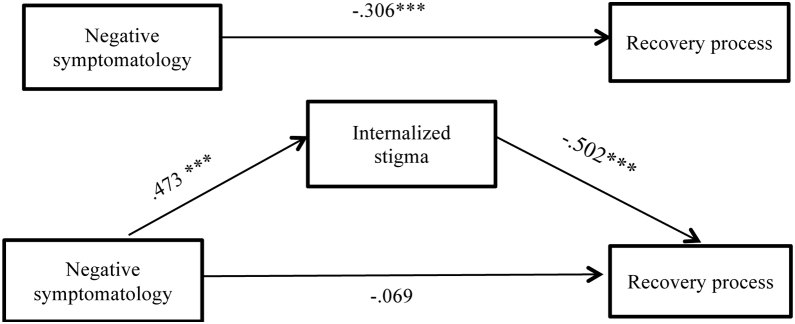
Results of the mediation model: Indirect effect of negative symptomatology on recovery of people with psychosis through internalisation of stigma and total effect (standardised regression coefficients). ***p ≤ .001.

In Figure 2, we can observe the results of the mediation model, which indicate that the indirect effect is significant.

The second aim of the study tries to analyze which stigma factors are mediating in the total effect between negative symptomatology and the recovery from psychosis. Table 3 shows the results of the indirect mediation routes of each one of the internalized factors of the Stigma.

**Table 3:** Mediation Model: The indirect effect of negative symptomatology on recovery mediating each one of the internalized stigma factors.

Indirect effect					*B*	*Boot SE*	*Boot 95% CI*	*Sobel Test (Z)*
Negative symptomatology	→	Alienation	→	Recovery	**-.17**	.065	[-.383, -.121]	-2.58*
Negative symptomatology	→	Approval of the stereotype	→	Recovery	.03	.054	[-.073, .140]	0.58
Negative symptomatology	→	Discriminatory experience	→	Recovery	-.04	.042	[-.139, .031]	-1.10
Negative symptomatology	→	Social isolation	→	Recovery	-.01	.049	[-.115, .081]	-0.30
Negative symptomatology	→	Resistance to stigma	→	Recovery	**-.05**	.031	[-.121, -.004]	1.69^+^

*Note.* Completely standardized indirect effect. *^+^p* < .10; *p < .05.

**Figure 3:**
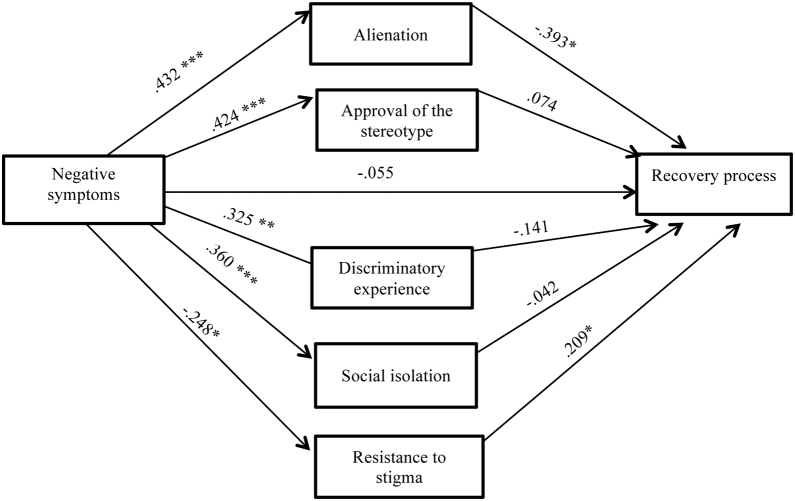
Results of the mediation model: The indirect effect of negative symptomatology on the recovery stages from psychosis through de dimensions of the internalised stigma construct (standardised regression coefficients). ***p ≤ .0001, **p ≤ .001; *p < .05.

As can be seen in Figure 3 only the indirect effects that have as mediating variables the dimensions of the internalised stigma Alienation and Resistance to stigma are significant.

Analysing the indirect effects, the results indicate that negative symptomatology predicts an increase in Alienation (*B* = 0.43, *p* < .0001) and this is what complicates the Recovery process (*B* = -0.39, *p* = .003). Along the same line, negative symptomatology predicts a decrease in Resistance to stigma (*B* = -0.24, *p* = .009) and this works as a protective factor or a boost to the Recovery process (*B* = -0.20, *p* = .021). Besides, the direct effect of negative symptomatology on the worsening of the Recovery disappears (*B* = -0.05, *p* = .552) if the mediation of the ISMI factors is kept in mind in this link.

The mediation of the Approval of stereotype, Discrimination experience, and Isolation social is not significant.

## Discussion

The main aim of this research study has been to confirm that the influence of negative symptomatology on the recovery from psychosis is not direct, but instead, it would be mediated by the perceived stigma of the person regarding his disorder. The results of the current report seem to point out that the influence of negative symptomatology predicts an increase in stigmatization perceived by the person due to his disorder, and this in turn, affects his recovery process negatively. This could have important repercussions on clinical practice, since the factors that affect the prognosis regarding personal recovery are well known, however the mechanisms and processes that ease this kind of recovery have been less studied (Bjornestad et al., 2017).

In a recent meta-analysis conducted by Devoe et al. (2018) they compare the effect of the NMDAR modulators (N-methyl-D-aspartate-receptor), omega-3, antipsychotics, psychosocial interventions, CTR (Cognitive Remediation Therapy), therapies based on necessity and integrated psychological therapies for negative symptomatology in individuals with high clinical psychosis risk. In this way, they discover that no treatment reduced significantly the negative symptoms. Thus, given the difficulties in the psychological and pharmaceutical approach to negative symptomatology, an intervention over the stigma itself should therefore, be explored as an alternative to trying to improve the recovery process of people.

On the other hand, there are many investigations in current literature that have made a direct link between greater presence of negative symptomatology and worse recovery. Recently, Norman et al. (2018) in a five-year longitudinal study, examined the parts implied in such a recovery process in people with psychosis, finding out that a less negative symptomatology was associated to a bigger recovery. In the same line, Austin et al. (2013) (carried through a meta-analysis on patients with a first episode of psychosis finding a significant link between the lesser gravity of negative symptoms and the best recovery rate in 10 years (odds ratio = 0.53, 95% IC = 0.36-0.78, *p* <  .001). In the current study, we have also confirmed the link between negative symptomatology and recovery of people with psychosis (Hypothesis 1: *B* = -0.30, *p* =  .001).

Nevertheless, as far as we can ascertain this is the first research study, as far as we know, which states that such a link is not direct, but instead, is explained by the total mediation of the stigma. That is, the prediction of negative symptomatology on the recovery process disappears when we include the stigma as a mediator variable. In other words, in the first place, we have discovered that negative symptomatology predicts an increase in stigma in people with psychosis. It has also been confirmed that this level of internalized stigma is that which precisely influences the recovery process negatively (and not negative symptomatology *per se*).

Regarding the role of negative symptomatology in stigma, the recent study by Gee et al. (2019), through a qualitative analysis, analyses the role negative symptoms play in 24 people with a first psychotic episode. Their results show that people with psychosis attribute negative symptomatology -like emotional degradation, apathy and unsociability- to the secondary effects of medication and lack of trust or social avoidance, to protect themselves from rejection linked to internalized stigma. In this direction, Horsselenberg et al. (2016) studied the link between the stigma and psychotic symptomatology in 102 people diagnosed with a type of schizophrenia spectrum disorder. Their results did show that the link between positive symptoms and stigma was mediated by victimization. Nevertheless, they stated a direct link between negative symptomatology and stigma.

In line with our approach, the previous investigation shows that internalized stigma influences the recovery process negatively. Reports similar to that of Oexle et al. (2018) also state such a link. Recently, Singla et al. (2020) have found similar results after checking that people who are in a higher phase of recovery, show a lower stigma. Given the importance that stigma appears to have in the recovery process, there are many recent studies that try to state possible mediating variables between stigma and recovery (Fowler et al., 2015, García-Mieres et al., 2020, Vass et al., 2017).

Furthermore, in the present study there has been an analysis of the factors of the internalized stigma which would be mediating between negative symptomatology and recovery from psychosis. Our results showed that the stigma factors Alienation and Resistance to stigma mediate in such a link: negative symptomatology would have some influence, increasing Alienation (experience of the person with psychosis of not being a full member of society or being lowly valued or excluded) and decreasing Resistance to stigma (the ability of not being affected by stigma) making the recovery process more difficult. Nevertheless, the fact that the individual experiencing psychosis agrees with stereotypes (Approval of stereotype), the way he is treated by others (Experience of discrimination) or avoids social contact for fear of rejection (Isolation social) does not seem to reveal worse recovery.

In conclusion, these results support the importance of including the reduction of the stigma as a specific strategy in order to improve the recovery process from psychotic disorders, specially focused in Alienation and in Resistance to stigma. These results support the inclusion of stigma reduction within the treatment of psychotic disorders along with other treatments or therapies that have already demonstrated their effectiveness, such as Mindfulness or the Metacognitive Insight and Reflection Therapy (López-Navarro et al., 2020, Lysaker et al., 2020). However, they should be taken into consideration in light of the following limitations: The sample was made up of people experiencing psychosis who function quite well, with a steady course and ambulatory care. This is not representative of everyone that has psychotic disorders and the results cannot be generalised to patients whose disorders could be presumably more severe. In the same way, no information from external sources that could increase and guarantee the accuracy of such information has been obtained, so the self-report measure such as internalized stigma may not capture other conceptualizations of these constructs. In addition, the sample is made up mainly of males (72%) and it is unclear whether these findings would be generalized to females. Nor have we included an assessment of positive symptoms of schizophrenia, which have been shown to be associated with internalized stigma (Lysaker et al., 2007, Park et al., 2013), so the relationship between internalized stigma and the full range of psychiatric symptoms could not be examined. Lastly, the cross-cutting nature of the study cannot state cause-effect inferences. In future studies, these results should be explored in longitudinal investigations of an experimental kind.

With regard to future investigations, other possible mediating variables that could influence the relationship between stigma and recovery and/or between negative symptomatology and recovery should be sought. Similarly, it should be explored wither similar results are found with the more pragmatic, sensitive and less rigid diagnostic criteria of ICD-11 (Medina-Mora et al., 2019), as well as what role positive symptoms (Lysaker et al., 2007, Park et al., 2013, Rector et al., 2005) or depression (Ritsher et al., 2003), that have been linked in the literature to internalized stigma, may play in our results.
